# Combination therapy with B7H3-redirected bispecific antibody and Sorafenib elicits enhanced synergistic antitumor efficacy

**DOI:** 10.7150/thno.49480

**Published:** 2020-08-21

**Authors:** Cheng Huang, Hongjian Li, Yunyu Feng, Xiaoling Li, Zongliang Zhang, Caiying Jiang, Jichao Wang, Chenli Yang, Yuying Fu, Min Mu, Shasha Zhao, Zeng Wang, Yi Kuang, Huan Hou, Yuelong Wang, Wenhao Guo, Jianguo Xu, Hui Yang, Liangxue Zhou, Aiping Tong, Gang Guo

**Affiliations:** 1 State Key Laboratory of Biotherapy and Cancer Center, West China Hospital, West China Medical School, Sichuan University, Chengdu, Sichuan province, China.; 2 Department of Neurosurgery, West China Hospital, West China Medical School, Sichuan University, Chengdu, Sichuan province, China.; 3 Department of Abdominal Oncology, West China Hospital, West China Medical School, Sichuan University, Chengdu, Sichuan province, 610041, China.; 4 Department of Otolaryngology, Head and Neck Surgery, West China Hospital, West China Medical School, Sichuan University, Chengdu, Sichuan province, 610041, China.

**Keywords:** Ovarian cancer, sorafenib, immunotherapy, B7H3, bispecific antibody

## Abstract

**Rationale:** Current traditional treatment options are frequently ineffective to fight against ovarian cancer due to late diagnosis and high recurrence. Therefore, there is a vital need for the development of novel therapeutic agents. B7H3, an immune checkpoint protein, is highly expressed in various cancers, representing it a promising target for cancer immunotherapy. Although targeting B7H3 by bispecific T cell-engaging antibodies (BiTE) has achieved successes in hematological malignancies during recent years, attempts to use them for the treatment of solid cancers are less favorable, in part due to the heterogeneity of tumors. Sorafenib is an unselective inhibitor of multiple kinases currently being tested in clinical trials for several tumors, including ovarian cancer which showed limited activity and inevitable side effect for ovarian cancer treatment. However, it is able to enhance antitumor immune response, which indicates sorafenib may improve the efficiency of immunotherapy.

**Methods:** We evaluated the expression of B7H3 in ovarian cancer using online database and validated its expression of tumor tissues by immunohistochemistry staining. Then, B7H3 expression and the effects of sorafenib on ovarian cancer cell lines were determined by flow cytometry. In addition, 2D and 3D ovarian cancer models were established to test the combined therapeutic effect *in vitro*. Finally, the efficiency of B7H3×CD3 BiTE alone and its combination with sorafenib were evaluated both *in vitro* and *in vivo*.

**Results:** Our data showed that B7H3 was highly expressed in ovarian cancer compared with normal samples. Treatment with sorafenib inhibited ovarian cancer cell proliferation and induced a noticeable upregulation of B7H3 expression level. Further study suggested that B7H3×CD3 BiTE was effective in mediating T cell killing to cancer cells. Combined treatment of sorafenib and B7H3×CD3 BiTE had synergistic anti-tumor effects in ovarian cancer models.

**Conclusions:** Overall, our study indicates that combination therapy with sorafenib and B7H3×CD3 BiTE may be a new therapeutic option for the further study of preclinical treatment of OC.

## Introduction

Ovarian cancer (OC) is the most fatal gynecologic malignancy 1, 2. Due to the complicate pathogenesis of OC, it is hard to diagnose at an early stage 3. The current approach to OC is surgery, chemotherapy and targeted therapy 4. Although most patients respond to primary treatment, they usually experience a recurrence of the disease, resulting in a dismal overall 5-year survival rate of 30% 5. Since OC prognosis is still poor, new treatment options are urgently needed.

Recently, immunotherapy has emerged as a beneficial tool for cancer therapy by activating the immune system to fight against tumor cells, including antibodies, vaccines and engineered cells 6-8. Among these treatments, bispecific T-cell-engaging antibody (BiTE), which promotes T cell infiltrating in tumors and facilitating the efficacy of tumor-killing, constitutes the most promising approach 9-11. Until now, BiTE has delivered an outstanding clinical performance in hematologic malignancies and several solid tumors 12. According to previous clinic studies, intraepithelial CD8+ T lymphocyte infiltration is closely related to favorable overall and progression-free survival, suggesting that T cells can play a key role in controlling tumor growth 13-15. These evidences suggest that OC may be amenable to BiTE.

Due to the potency of BiTE, it is crucial to select a tumor-associated antigen with low toxicity and high selectivity. B7H3 belongs to the B7 family of immune checkpoint proteins 16. Aberrant expression of B7H3 has been found in a variety of cancers, such as melanoma, head and neck cancer and OC. Previous studies have demonstrated that B7H3 is responsible for invasion, migration and proliferation of tumor cells and drug resistance 17-19. Blockade of B7H3 by chimeric antigen receptor T cells or monoclonal antibody has shown ideal outcomes for other types of cancer, including OC in preclinic researches 20-22. Therefore, B7H3 is considered as a promising target for the treatment of OC.

Sorafenib is a small molecule multitargeted kinase inhibitor that blocks the activation of RAF, VEGF receptor and platelet-derived growth factor receptor β 23. Among these targets, RAS signaling pathway plays a central role in OC development 24. Preclinical studies have indicated that sorafenib is effective in a broad spectrum of tumor types, including renal cell carcinoma, hepatocellular carcinoma and OC 25. Clinic studies have found a modest anti-tumor effect for patients with OC by sorafenib treatment, with the expense of significant toxicity 26. Besides, recent findings have revealed that sorafenib abrogates the immunosuppression in patients with several types of tumors 25,28,29. Therefore, combination with sorafenib and immunotherapy is probably an attractive treatment modality.

In this study, a B7H3×CD3 BiTE is developed and the potential cytotoxicity against OC is accessed. In addition, the influence of sorafenib on OC growth and its expression level of B7H3 are also explored. Finally, a combination treatment regimen is established to evaluate the efficiency *in vitro* and *in vivo*.

## Material and Methods

### Animals

8-10-week-old immunodeficient female NSG mice were obtained from the Beijing Huafukang Biotechnology Co. Ltd (Beijing Huafukang Biotechnology Co. Ltd, Beijing, China) and housed in a specific pathogen free environment at Sichuan University. The animal experiments were approved by the Biomedical Research Ethics Committee at West China Hospital (Ethical approval document: 2018-061). All efforts were made to minimize suffering.

### Cell culture

The human OC cell line SKOV3, H8910, A2780, OVCAR-3, A375 and Hela were obtained from American Type Culture Collection and maintained in DMEM (Gibco) supplemented with 10% fetal bovine serum and 2 mmol/L L-glutamine (Invitrogen), 1% penicillin and streptomycin.

Human peripheral blood mononuclear cells (PBMC) were isolated from healthy donors (informed written consent from all participants was obtained prior to the research) following Ficoll density gradient centrifugation (800 ×g for 15 min at room temperature). The experiment was approved by the Biomedical Research Ethics Committee at West China Hospital (Ethical approval document: 2018-061). Lymphocytes were isolated from whole blood using Lymphoprep (Greiner Bio-One) and cultured in X-vivo medium (Lonza) supplemented with 10% fetal bovine serum (heat inactivation at 56 ℃ for 30 min), 2 mM L-glutamine and 100 U/mL penicillin. PMBC were stimulated with CD3 monoclonal antibody (mAb) (OKT3, 200 ng/mL, BioLegend), CD28 mAb (CD28.2, 100 ng/mL, BioLegend) for three days. Then continuous culture was performed using recombinant human IL-2 (100 units/mL, Life Science). All cells were collected in a logarithmic growth phase and were used for all *in vitro* and *in vivo* experiments in 5% CO_2_ at 37 °C.

### Construction of B7H3×CD3 BiTE

The anti-B7H3 single-chain variable fragment (scFv) sequence used in BiTE was derived from a highly specific mAb against B7H3 (clone mAb-J42) generated by our lab group using a standard hybridoma technique. cDNA encoding the CD3-specific scFv (according to published amino acid sequences, see detailed sequence in supplementary information) and B7H3-specific scFv (J42-scFv) were synthesized by Genewiz. A recombinant single-chain BiTE was formed by linking a G4S linker between the two scFvs. The recombinant cDNA was subcloned into a eukaryotic expression vector with a His tag at the C-terminal to facilitate protein purification.

HEK293T cells were transduced with the expression vectors described above and cultured in FreeStyle serum-free medium (Thermo Fisher Scientific) at 37 °C with 5% CO_2_ in a humidified incubator. After one week, culture supernatant was harvested and pre-cleaned by 0.22 μM filters. Then the recombinant B7H3×CD3 BiTE was purified on Ni-NTA affinity columns and subsequently subjected to carry out fine purification by using Superdex 200 increase 10/300 GL Column (GE). Purified BiTE was routinely analyzed by SDS-PAGE and stained with Coomassie brilliant blue for size estimation and quality control. HEK293T cells were transduced with the expression vectors described above and cultured in FreeStyle serum-free medium (Thermo Fisher Scientific, Waltham, MA, USA) at 37 °C with 5% CO_2_ in a humidified incubator. After one week, culture supernatant was harvested and pre-cleaned by 0.22 μM filters. Then the recombinant B7H3×CD3 BiTE was purified on Ni-NTA affinity columns and subsequently subjected to carry out fine purification by using Superdex 200 increase 10/300 GL Column (GE). Purified BiTE was routinely analyzed by SDS-PAGE and stained with Coomassie brilliant blue for size estimation and quality control.

### Lentivirus transfection

Lentivirus transfection was used to establish OC cell lines that were marked with luciferase. Briefly, the luciferase viruses were produced by transfecting the luciferase plasmid along with packaging plasmids (psPAX2 and pMD2.G vectors). The lentivirus-infected SKOV3 cells were selected by 4 μg/mL puromycin for 7 days and stabilized by culturing for 4 weeks. Then, SKOV3-lucf stable cell line was finally obtained.

### Flow cytometry

B7H3 expression on tumor cell lines and tumors from mice was tested by FACS. After tumor-bearing mice received treatment, tumor tissues were harvested from tumor-bearing mice. Tissue was minced by scissors and incubated with digestion mixture (collagenase: 1 mg/mL) at 37 °C/30 min. The washing and centrifugation were repeated and then the cells were made into a single cell suspension. Tumor cell suspension was incubated with the human B7H3 antibody (BioLegend, 331605) and CD3 (BioLegend, 300311) for flow cytometric analysis (ACEA Bioscience) according to the manufacturer's protocols. Similarly, to detect the B7H3 expression on various cell lines, including SKOV3, H8910, A2780, OVCAR-3, A375 and Hela, flow cytometric analyses were performed. To assess the effect of SOR on T cell proliferation, cells were prelabeled with Cell Trace Cyto Tell Red (AAT Bioquest, 22255) and then flow cytometry analyses were utilized. For T cell phenotype analyses, total cells were stained with antibodies for surface expression of human CD25 (BioLegend, 302629), CD69 (BioLegend, 310909), CD4 (BioLegend, 357419) and CD8 (BioLegend, 344729) and analyzed by a Fortessa flow cytometer (BD). For apoptosis detection, Annexin V staining was performed using FITC-annexin-V Apoptosis Detection Kit I (4A Biotech). Briefly, SKOV3 cells (5×10^5^) were treated for 48 h with 10 μM sorafenib (MCE) and made into cell suspension. According to the manufacturer's protocols, samples were detected on a NovoCyte™ Flow Cytometer (ACEA Bioscience).

### Western blotting

After treatment with the indicated concentrations of SOR for 48 h, SKOV3 cells were lysed with RIPA buffer (Beyotime) supplemented with proteinase and phosphatase inhibitors (Sigma). The total protein extracts were then quantified with the BCA kit (Beyotime). All above mentioned procedures were carried out on ice. Then, proteins of equal quantities were subjected to 10% SDS/PAGE gels and transferred to polyvinylidene difluoride membranes. After that, the membrane was blocked with non-fat skimmed milk for an hour at room temperature before probing it with the different primary antibodies, including P-ERK1/2 (CST 4370S), P-MEK1/2 (CST, 3958S), B7H3 (CST, 14058S) and β-actin (ZSGB-BIO, TA09) antibodies. HRP-conjugated secondary antibody (Beyotime, A0208) for 1 h. Images were captured by a ChemiScope 6000 Touch (Clinx).

### Cytotoxicity assays *in vitro*

For tumor cell proliferation analysis, cells were seeded in 96-well plates and treated with various concentrations of SOR after 72 h. Cell Counting Kit-8 solution (Beyotime) was added and incubated in 5% CO_2_ at 37 °C. The result was measured in a microplate reader at 450 nm. T cell cytotoxicity was measured by chromium-51 (51Cr)-release assays using the procedure reported previously 27. Two-dimensional and three-dimensional tumor models with T cells were used to assess the cytotoxicity and observe cell morphology. In the two-dimensional model, SKOV3 cells were co-cultured with T cells at an E:T ratio of 1:5, 1:1 and 5:1, together with 5 μM SOR alone or in combination with 5 μg/mL BiTE. Images were captured at 24 h after co-culturation. In order to better observe the morphology of tumor cell lysis. The fluorescent dyes Cyto Tell Red and CFSE (Beyotime) were used to stain target cells (SKOV3) and effector cells (T cell) (E:T=5:1), respectively. The SKOV3 and T cell were co-cultured for 24 h and visualized by confocal laser scanning microscope (Zeiss 880). To develop a three-dimensional model, SKOV3 cells (5×104 cells /mL) were seeded in ultra-low attachment dishes (Corning, USA) and grown in serum-free DMEM/F12 medium (Gbico) supplemented with 2% hormone mixture B27 (Gibco, USA), 20 ng/mL human recombinant epidermal growth factor (Sino Biological), 10 ng/mL human recombinant fibroblast growth factor 2 (Sino Biological) and 1 μg/mL heparin for 7-14 days. Fresh medium was added to the culture every 48 or 72 h. After staining with CFSE (Beyotime), T cells were added to the wells at the E:T ratio of 1:1 with 5 μg/mL BiTE alone or in combination with 5 μM SOR for 12 h. Images were captured on a fluorescence microscopy.

### Analysis of cytokine secretion

Target cells were co-cultured with effect cells (E:T ratio, 5:1,1:1,1:5) in a 96-well plate at the temperature of 37 °C with an addition of different concentration of BiTE. After 24 h, the supernatant was collected to analyze the IFN-γ secretion from the effector cells using ELISA kit (Thermo Fisher Scientific) according to the manufacturer's protocols.

### Real-time monitoring of cytotoxicity

This assay utilized the xCELLigence® real-time cells analyzer (ACEA Biosciences, Inc.) to monitor the cytotoxicity of the indicated treatment groups. Briefly, background readings from 50 μL of culture medium added in each well of the E-plate® 96 were obtained. The SKOV3 cells were added into the wells at a density of 8×10^3^ cells/well (optimized cell density), and the plate was equilibrated at RT for 15 min before placing in the RTCA-SP station. In order for the cells to attach to the E-plates, the cells were cultured in 5% CO_2_ incubator for 15 h (optimized duration). 15 h later, SOR (5 μM), BiTE (5 ng/mL) and T cell suspensions (E:T=5:1) were added into the specific well. Three replicates were available for each well. The SOR were dissolved in DMSO with a final concentration ≤1.0% in the cell culture. Vehicle control cultures received DMSO alone. Cell index measurements were performed at 15 min intervals for 72 h.

### Animal studies and bioluminescent imaging (BLI)

In the SKOV3 xenograft experiments, we established two xenograft tumor models of subcutaneous and intraperitoneal tumors. Cells were collected in a logarithmic growth phase and inoculated as soon as possible. 5×10^6^ or 1×10^7^ SKOV3 cells were intraperitoneally or subcutaneously injected into female NSG mice, respectively. From the eight day on, the drug treatment groups were administered with sorafenib by oral gavage at 20 mg/kg for 15 consecutive days. The BiTE treatment group was treated with 1×10^7^ T cells and 100U IL-2 or in combination with 3 mg/kg BiTE per mouse on day 11, 15 and 19, respectively. The mice in the combination group were treated with both sorafenib and BiTE as described above. Body weight and tumor sizes were measured every three days. Tumors size were measured every 3 days by a slide caliper, and tumor volume was calculated by the formula: Volume = Length×Width×Height/2.

The BLI was conducted using an *in vivo* imaging system (Caliper Life Sciences). A 150 mg/kg dose of D-Luciferin potassium salt (MeiLunBio) was intraperitoneally injected into the mice 10 min before imaging. The region of interest (ROI) was selected and the radiance value was measured by Living Image® 4.3.1 Software (https://www.perkinelmer.com). At the end-point of observation, all the mice were euthanized immediately after the last *in vivo* BLI.

### Immunofluorescence and IHC staining

SKOV3, A375 and HeLa cells were cultured (4×10^4^ cells/well) in 24-well plates, and incubated at 37 °C in a 5% CO_2_ incubator overnight. Then cells were blocked with 5% BSA for 15 min and stained with B7H3 antibody (Abcam, ab227679) at 4 °C overnight. Cells were incubated with Cy3-conjugated secondary antibody (1:200; Proteintech) at room temperature in the dark. After staining with 4,6-diamidino-2-phenylindole (DAPI) (Beyotime) away from light for 3 min, the images were captured on a confocal laser scanning microscope (Zeiss 880).

Human tumor tissue microarrays for IHC were purchased from Xi'an Alenabio and Shanghai Outdo Biotech of China. Next, the expression of microenvironment-related markers in tumor tissues was detected by immunohistochemistry, including CD3 (Servicebio, GB13014), CD4 (Servicebio, GB13064-2), CD8 (Servicebio, GB13429), CD31 (Servicebio, GB11063), Ki67 (Servicebio, GB13030-2) and B7H3 (CST 14058S). For the analysis of all markers, areas of acute inflammation and necrosis were avoided. Photomicrographs were taken of sixteen areas enriched for intraepithelial CD3+, CD4+ and CD8+ lymphocytes with blinding to mutational status. Tumor cell proliferation and angiogenesis was determined by nuclear Ki67 staining and CD31. In brief, 3-4-mm-thick tissue sections were incubated at 65 °C for 1 h to retrieve antigenicity, then blocked with phosphate-buffered saline (PBS) containing 10% normal goat serum (Boster) for 30 min at room temperature. Next, the tissue sections were incubated with primary antibody at 4 ℃ overnight and then incubated with secondary antibodies. The fluorescence IHC staining manipulation followed the same steps as the IHC assay. The only difference was that fluorescent second antibody (Servicebio, GB213031) was added. Pictures of stained sections were captured using a Digital Pathology System (Pannoramic MIDI, 3DHISTECH, Hungary).

### Statistical analysis

The relationship between CD276 gene expression and prognosis was performed using the OC dataset of the KM Plotter. B7H3 mRNA expression, the tumor stage and grade of OV patients were examined by dada mining in TCGA-OV using the UCSC xena browser. The meta-analysis was performed by using Oncomine. Boxplots with significance levels were plotted using the “ggpubr” package version 0.2.4 in R language (version 3.6.1). Data were evaluated using the Student's t test to determine the statistical differences between various experimental and control groups. Statistical analyses were performed using GraphPad Prism 6.0 and considered significant at *P < 0.05; **P < 0.01; ***P < 0.001.

## Results

### Analysis of B7H3 expression based on online database

**Figure 1:**
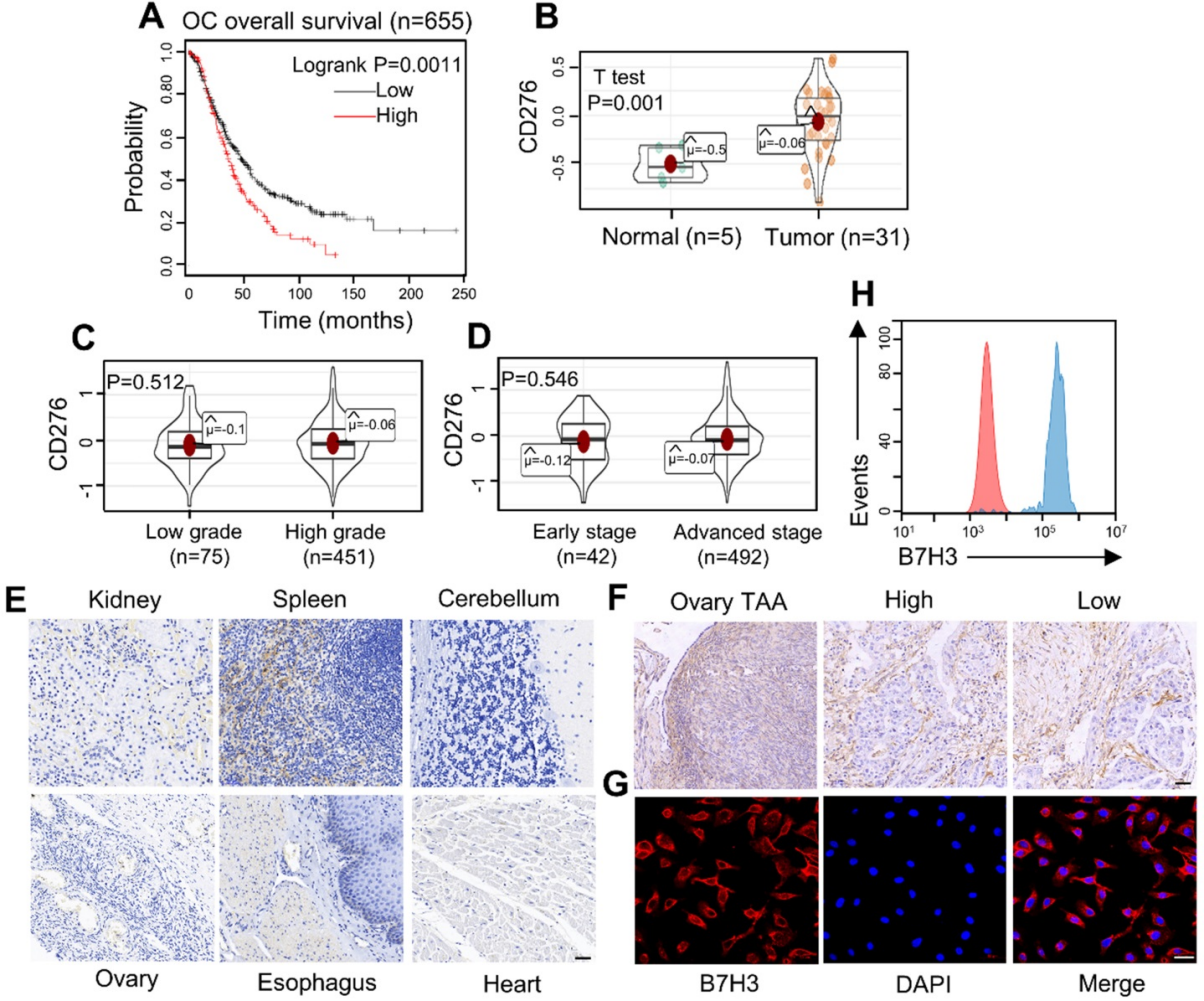
** Expression of the costimulatory molecule B7H3 in human OC.** (**A**) Relationship between B7H3 expression level and overall survival of patients with OC. (**B**) The mRNA expression of CD276 in the tumor and normal ovarian tissue samples analyzed with the TCGA database. (OC vs. normal ovaries, *P <0.05). (**C, D**) The expression level of B7H3 mRNA expression. B7H3 mRNA expression stratified according to (C) tumor grade (D) tumor stage (P>0.05). (**E**) Microarrays of human normal tissues were stained for IHC to detect the expression of B7H3. Scale bar, 50 µm. (**F**) Microarrays of human ovarian tumor-adjacent tissues and OC tissues were stained for IHC to detect the expression of B7H3. High, high grade. Low, low grade. Scale bar, 50 µm. (**G, H**) Flow cytometry and immunofluorescence staining patterns showed high expression of the B7H3 in OC cell line (SKOV3). Scale bar, 50 µm.

Based on KM Plotter database, we analyzed the overall survival significance by comparing carcinomas with high B7H3 levels to those with low B7H3 expression (Figure 1A). The low-B7H3 group showed significantly better overall survival than the high-B7H3 group (log-rank test, *p =* 0.0011). Then, the mRNA expression level of B7H3 was tested in normal and tumor samples (Figure 1B). It was found that B7H3 was significantly higher in OC than that in the normal groups (*p =* 0.001). In addition, association between B7H3 mRNA expression levels and stage (Early stage: clinical stage I, II. advanced stage: clinical stage III, IV) or grade (Low grade: G1, G2. High grade: G3, G4) in OC patients were evaluated (Figure 1C, D). Statistical analysis indicated no significant correlation between different groups. Finally, four studies showing the differential expression of B7H3 in OC tissues compared with normal tissues. Darker red indicates higher B7H3 expression (Figure S1A). As a result, we considered B7H3 as a biosafe clinical target.

### Frequent expression of B7H3 in diverse ovarian cancer cell lines and tumor tissues

Immunohistochemistry (IHC) was carried out to analyze the expression of B7H3 in tissue microarrays (Figure 1E and 1F). The intensity of B7H3 expression increased obviously in malignant tumor and tumor-adjacent tissues compared to normal tissues. Also, the expression level of B7H3 in various cancer cell lines were analyzed by flow cytometry using fluorescence-activated cell sorting (FACS) and immunofluorescence (Figure 1G, 1H and Figure S1B-D). The data showed that all tumor cell lines had a high level of B7H3 expression. Together, these results indicate that B7H3 may serve as a promising clinical target for OC treatment.

### Multi-kinase inhibitor SOR inhibits cell growth in SKOV3 cell and may involve an up-regulation of B7H3

**Figure 2:**
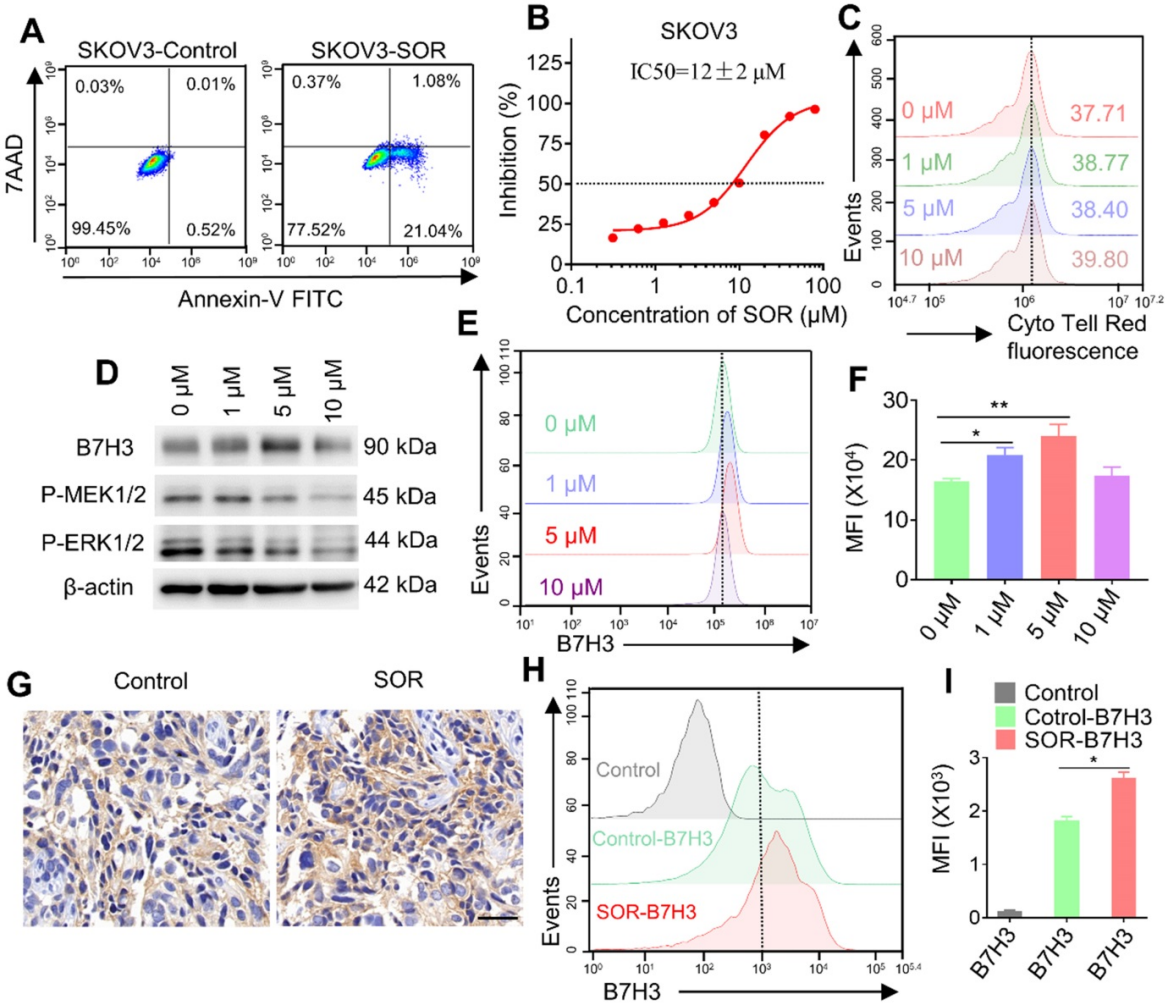
** Impact of multi-kinase inhibitor SOR in human T cells, tumor cells and mouse tumor model.** (**A**) Apoptosis was evaluated using an annexin V-FITC apoptosis detection kit and flow cytometry. The representative pictures are from SKOV3 cells cultured with 10 µM SOR. (**B**) SKOV3 cells were treated with various doses of SOR (0-80 µM) for 72 h, and IC50's of SOR was assessed by the Cell Counting Kit 8 (CCK-8) assay. (**C**) The effect of SOR on T cell proliferation. T cells were prelabeled with Cell Trace Cyto Tell Red and then treated with different concentrations of sorafenib for 48 h. (**D**) Analysis of B7H3, P-MEK and P-ERK in SKOV3 cells after treatment with indicated drug concentrations *in vitro*. (**E**) Cells with the same treatment were digested and stained with PE-conjugated B7H3 antibody and were analyzed using a flow cytometer. (**F**) Mean fluorescence intensity histogram for B7H3 in figure E. *P < 0.05, **P < 0.01. (**G**) *In vivo* analysis of B7H3 in SKOV3 xenograft tumor model by IHC. Scale bar, 20 µm. (**H**) Tumor with the same treatment were digested and stained with PE-conjugated B7H3 antibody and were analyzed by flow cytometry (Control, Control-B7H3, SOR-B7H3). (**I**) Mean fluorescence intensity histogram for B7H3 figure H. *P < 0.05.

To evaluate the effect of multi-kinase inhibitor SOR on SKOV3 cells and T cells. Tumor cells apoptosis and proliferation were analyzed by flow cytometry and CCK-8 analysis. The result of tumor cell apoptosis revealed that the apoptosis rate in the SOR (10 μM) treatment group increased (approximately 20%) compared with the control/DMSO group (Figure 2A). Similarly, SOR had a dose-dependent killing effect on SKOV3 cells, and the IC50 of SOR on SKOV3 cells was 12 ± 2 μM (Figure 2B). In addition, there is no evidence showed that SOR suppressed the growth of human T cells by Cell Trace Cyto Tell Red staining (Figure 2C). During this process, an interesting phenomenon was observed that SOR might affect B7H3 expression in SKOV3 cell lines. Tumor cells were incubated with rising concentrations of SOR for 48 h and the B7H3 protein expression level was determined by western blot and flow cytometry (Figure 2D-F). Interestingly, B7H3 protein expression was significantly increased following the application of SOR 1-5 μM, and returned toward the basal level when the drug concentration was raised to 10 μM. Thus, SOR concentrations ≤5 µM were selected for all subsequent experiments *in vitro*. The B7H3 expression in animal experiments was further tested. One week after mice were inoculated with 1×10^7^ SKOV3 cells in the right flank, they were orally gavaged with SOR at 20 mg/kg for 15 consecutive days. The data also revealed an upregulated B7H3, which is consistent with the *in vitro* results (Figure 2G-I).

### Characterization and functional test of B7H3×CD3 BiTE

**Figure 3:**
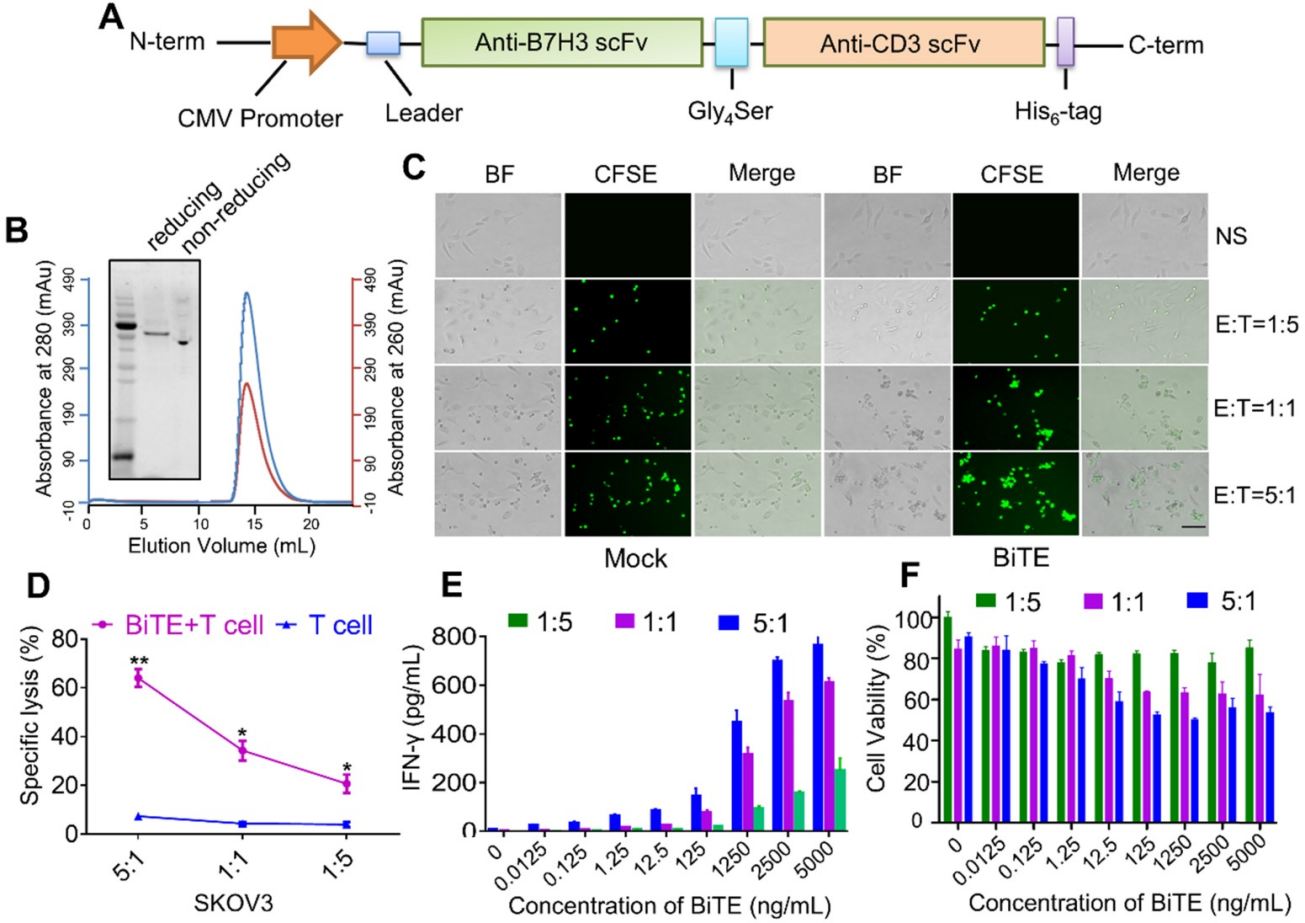
** Construction and cytotoxicity of anti-B7H3×CD3 BiTE *in vitro*.** (**A**) Schematic diagram of B7H3×CD3 BiTE construction. (**B**) The SDS PAGE and Gel filtration chromatograph of anti-B7H3×CD3 BiTE. (**C**) Morphology of tumor cells after incubated with human T cells (n = 3 donors) labeled with Cell Tracker CFSE at indicated ratios. Group “BiTE” was treated with anti-B7H3×CD3 BiTE at a concentration of 5 µg/mL. Scale bar, 100 µm. (**D**) ^51^Cr-release assays of anti-B7H3×CD3 BiTE against SKOV3 cell line at different E:T ratios. Group “BiTE” was treated with anti-B7H3×CD3 BiTE at a concentration of 5 µg/mL. *P < 0.05, **P < 0.01. (**E**) T cells co-cultured with target cells at various anti-B7H3×CD3 BiTE concentrations and E:T ratios for 24 h. The IFN-γ secretion levels were measured by ELISA kit. (**F**) Cell viability of SKOV3 cells after treatment with different concentrations of anti-B7H3×CD3 BiTE and E:T ratios for 12 h. Cell viability was measured using Cell Counting Kit-8 assays.

B7H3×CD3 BiTE was engineered by combining a B7H3 scFv derived from the mAb-J42 clone with a CD3 scFv. Each scFv contained a corresponding light chain (VL) and heavy chain (VH) joined together by a 5-amino-acid (G4S) linker (Figure 3A). Figure 3B showed the SDS-PAGE analysis of purified B7H3×CD3 BiTE by Gel filtration chromatograph on a superdex 200 increase 10/300GL column. In addition, coculture assay was carried out to assess the cytotoxicity of the BiTE towards cancer cell lines. More clusters of T cells and the lysis of cancer cells were observed in the BiTE groups compared to mock groups (Figure 3C). Next, the cytotoxicity of SKOV3 cells targeted by the B7H3×CD3 BiTE at various E:T ratios was tested by ^51^Cr release assays. Specific lysis was observed at 4 h (Figure 3D). By contrast, no significant response was observed in groups without the BiTE. Furthermore, IFN-γ secretion was measured by using an ELISA kit. Concomitant with this increase in concentration of BiTE, an increasing secretion of the IFN-γ cytokine can be observed. The group at 5:1 (E:T) ratio exhibited the highest cytokine levels (Figure 3E). Meanwhile, CCK-8 assay showed that SKOV3 cell viability decreased significantly with the increase in B7H3×CD3 BiTE concentration and E:T ratios (Figure 3F).

### Antitumor activity of B7H3×CD3 BiTE in combination with SOR are superior to single agents *in vitro*

**Figure 4:**
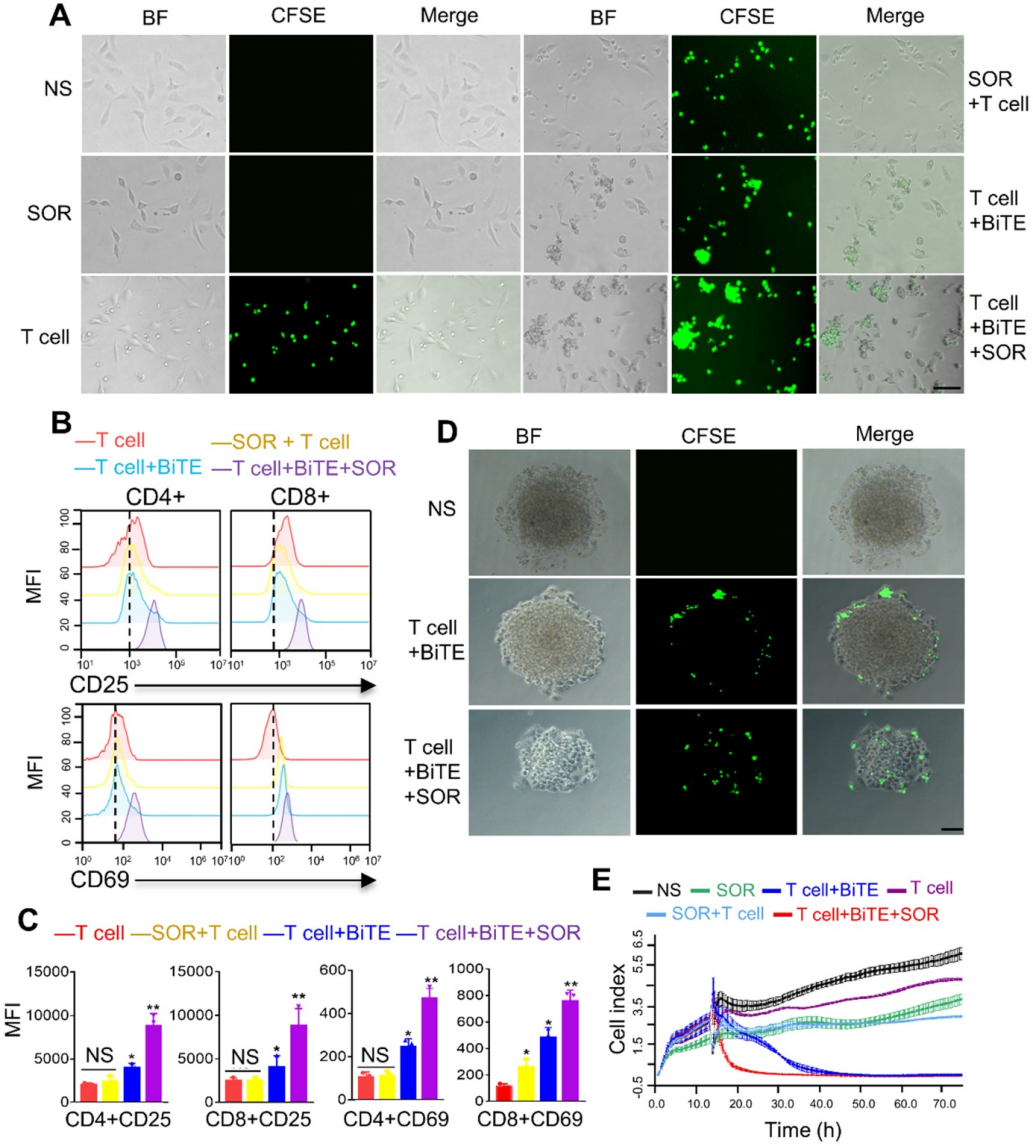
** Antitumor activity by anti-B7H3×CD3 BiTE in combination with SOR *in vitro*.** (**A**) Morphology of tumor cells after different treatments for 24 h. Scare bar, 100 µm. (**B**) Normal activation marker CD69 and CD25 expression on T cells. Human T cells were co-cultured with SKOV3 by adding 5 µM SOR alone or in combination with 5 µg/mL anti-B7H3×CD3 BiTE. T cells were collected 24 h and measured by 3-color flow cytometry. (**C**) Statistical analysis of the mean fluorescence intensity in figure B. *P < 0.05, **P < 0.01. (**D**) Infiltration and killing activity of T cells was detected using the 3D tumorsphere model after various treatments. SKOV3 tumoursphere were cocultured with T cells labelled CFSE. Scare bar, 50 µm. (**E**) Effect of different treatments on real-time cell cytotoxicity analyzer curves generated with SKOV3 cells.

In order to evaluate the synergism of B7H3×CD3 BiTE combined with SOR, coculture assay was carried out *in vitro*. Collectively, more T-cell clusters and cancer cell lysis were observed in the combination treatment group, demonstrating the synergistic antitumor effect with SOR and B7H3×CD3 BiTE (Figure 4A, Figure S3). This result was further supported by flow cytometric analysis of the expression of CD69 and CD25, two cell-surface markers depicting T cell activation. The expression levels were upregulated after combining with BiTE alone or in combination with SOR compared to T cell and SOR + T cell groups (Figure 4B, 4C). Meanwhile, the frequencies of CD4 and CD8 positive T cells subsets were analyzed in Figure S2. There was no obvious change throughout the coculture assay. In addition, we also established a three-dimensional (3D) spheroids model to simulate *in vivo* conditions. Although the phenomenon was found in treatment groups with the BiTE that T cell deposited within the peripheral rim of SKOV3 tumor regions, infiltration of tumor spheroids increased and the killing effect on the tumor was stronger in the combination therapy group (Figure 4D). The cytotoxicity of multiple treatment groups on SKOV3 cells were monitored with Real Time Cellular Analysis (RTCA) system and displayed as Figure 4E. The most synergistic effect was observed at the combination treatment group. Similarly, the antitumor activity of BiTE+T cell treated group had a stronger inhibition effects compared to that of the SOR, SOR+T cells and control group. In addition, no significant difference was observed in growth and proliferation between SOR and SOR+T cell.

### Combination of B7H3×CD3 BiTE and SOR synergistically inhibited tumor growth in OC subcutaneous tumor model

**Figure 5:**
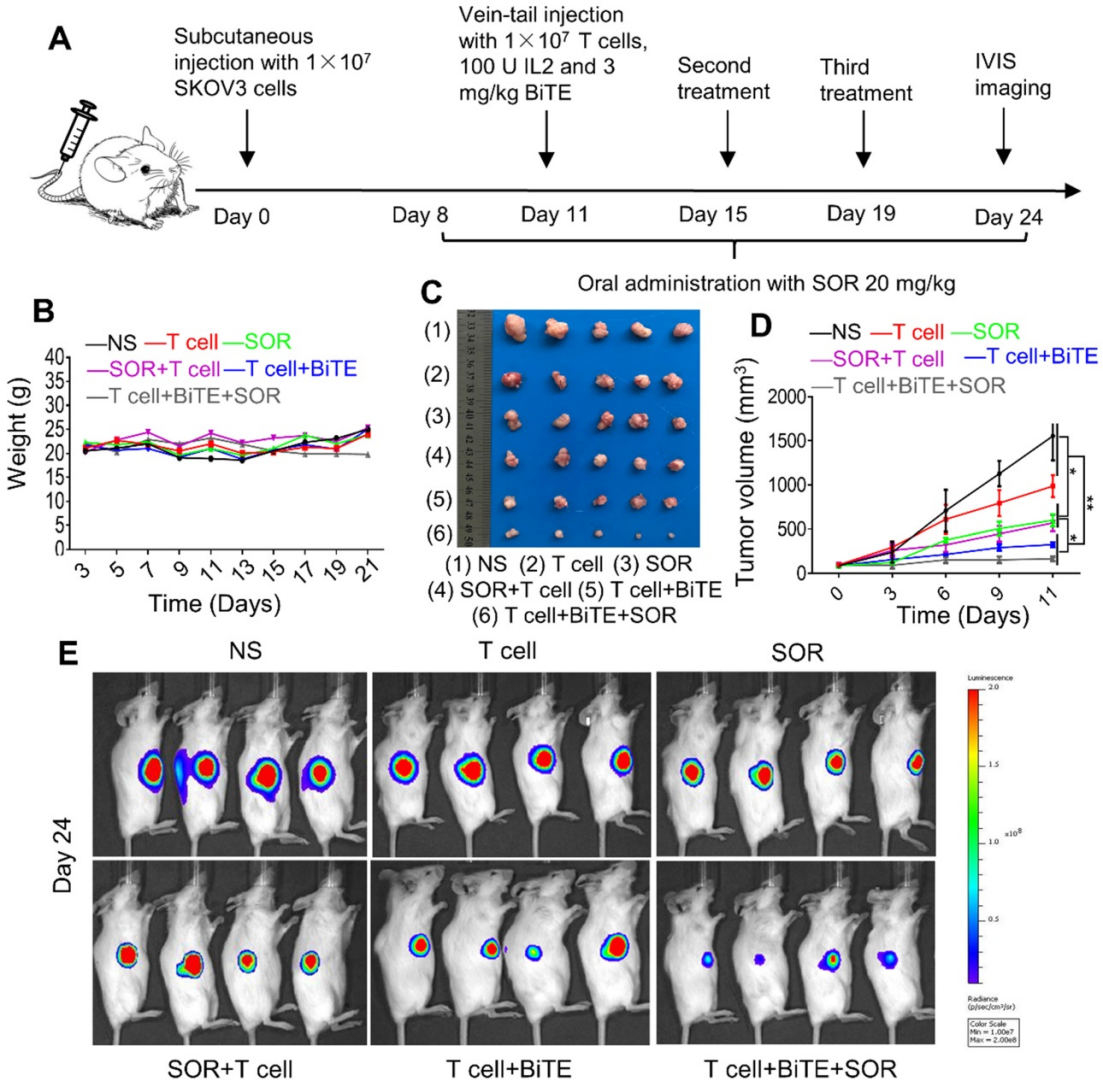
** Combination of multi‐kinase inhibitors SOR with anti-B7H3×CD3 BiTE therapy in human OC Subcutaneous Tumor Xenograft Models was superior to single therapy.** (**A**) The 5×10^6^ SKOV3-Luc cells were subcutaneously injected into NSG mice. Then the mice were treated with anti-B7H3×CD3 BiTE or SOR as described in the legend to figure A. (**B, C**) Body weight change (B) and photographs (C) from SKOV3 tumor-bearing mice on day 24 after the indicated treatments. (**D**) SKOV3 tumor growth curves after different therapies. *P < 0.05, **P < 0.01. (**E**) The representative tumor growth was shown *in vivo* by bioluminescence imaging using IVIS 200 on the 24th day.

To evaluate the antitumor effect of the combination treatment *in vivo*, a tumor xenograft model was established. Schematic diagram showed the *in vivo* treatment program (Figure 5A). After subcutaneous injection with SKOV3-Lucf cells, the mice were treated with indicated doses of T cells, SOR, SOR+T cells, T cells+BiTE or T cells+BiTE+SOR at indicated time points. Animal weights from each treatment group did not change significantly during the course of treatment (Figure 5B). As presented in Figure 5C, 5D, tumor volume was reduced significantly by SOR and SOR+T cells treatment, while the T cells+BiTE and T cells+BiTE+SOR treatment led to more obvious inhibition effects on the tumor volumes, compared to other groups. We also monitored the tumor growth by bioluminescence (Figure 5E). On day 24, the smallest fluorescence intensity was shown in mice treated with the T cells+BiTE+SOR, followed by tumors treated with the T cells+BiTE. This result is consistent with the solid tumor graph in Figure 5C.

### Combination of B7H3×CD3 BiTE and SOR synergistically inhibited tumor growth in OC intraperitoneal tumor xenograft models

**Figure 6:**
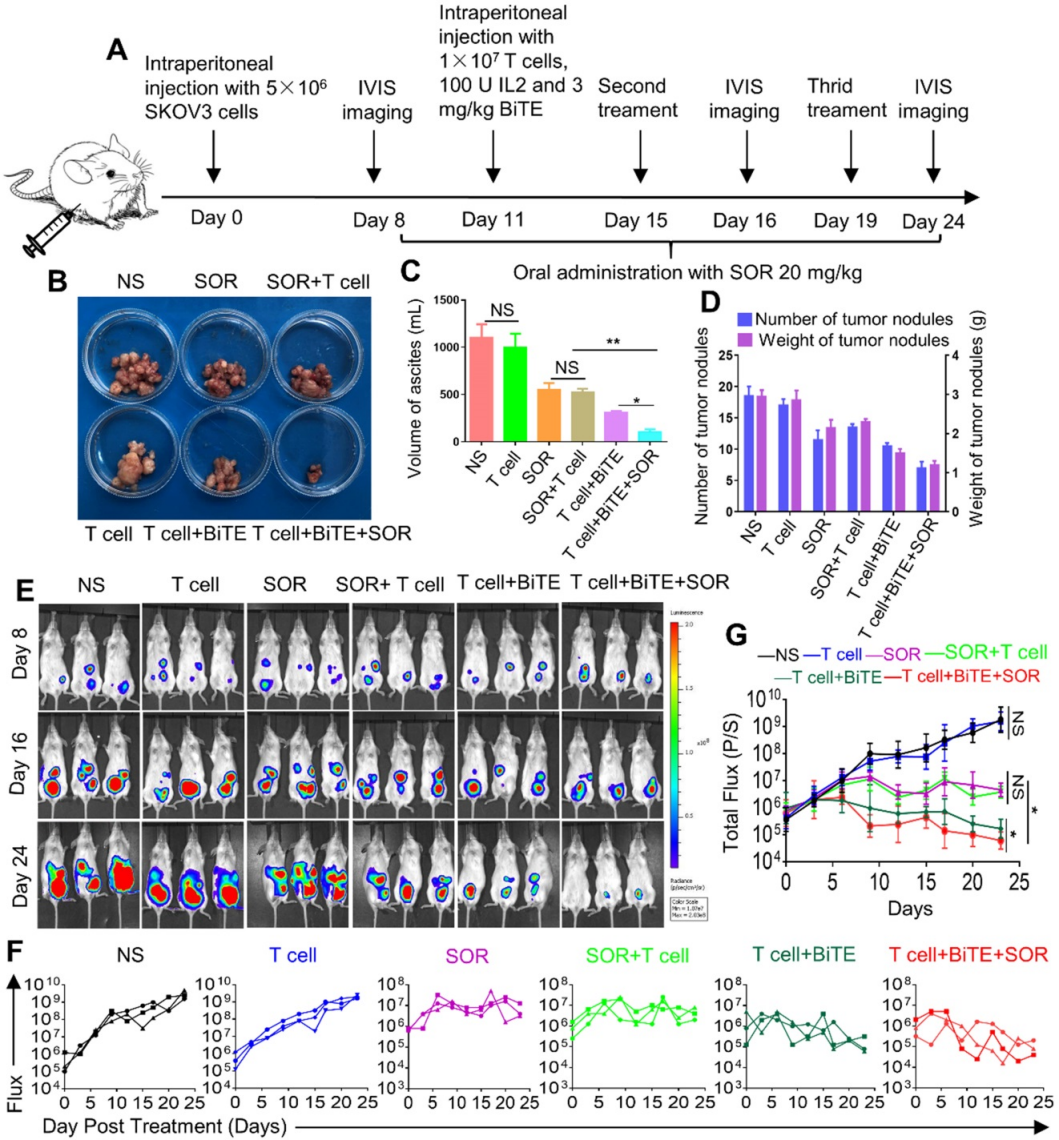
***In vivo* anti‐tumor effect of anti-B7H3×CD3 BiTE in combination with multi‐kinase inhibitors SOR against in human OC intraperitoneal tumor xenograft models.** (**A**) Schematic illustration showing the design of anti-B7H3×CD3 BiTE and SOR combination therapy as described above. (**B, C, D**) photographs (B), the volume of peritoneal ascites (C) and number and weight of tumor nodules (D) from SKOV3 tumor-bearing mice on day 24 after the indicated treatments. *P < 0.05, **P < 0.01. (**E**) Bioluminescence analysis of different treatments tumor growth over time; n=3. (**F, G**) Tumor total or individual flux data (in p/s) were calculated using Living Image software. Tumor growth rates are shown as mean values. *P < 0.05.

Although subcutaneous tumors are more easily and widely used for preclinical studies, intraperitoneal models implanted in physiologically relevant sites have more biological and pharmacological relevance to human disease. Schematic diagram illustrated the design of animal experiments (Figure 6A). The images of tumor nodes from the abdominal cavity were showed in Figure 6B. Furthermore, the ascites volume was collected from abdominal cavity and then measured. The BiTE+T cell and BiTE+T cell+SOR treatment reduced the volume of ascites compared with the other groups (Figure 6C). After sacrifice, the number of tumor nodes in the BiTE+T cell and BiTE+T cell +SOR treatment groups were significantly less than in the other groups, so was the weight of tumor nodes (Figure 6D). Bioluminescence imaging of tumor growth was carried out (Figure 6E). Quantification of the bioluminescence intensity of tumor foci in the abdominal cavity of animals was conducted after the course of treatment (Figure 6F, G). As is shown, significant antitumor activity was observed in the BiTE+T cell and BiTE+T cell+SOR treatment groups. Conversely, mice in the NS and T cell groups demonstrated progressive tumor burdens compared with the SOR and SOR+T cell treatment groups.

### Combination therapy of B7H3×CD3 BiTE and SOR increased T cell infiltration in OC subcutaneous tumor model

**Figure 7:**
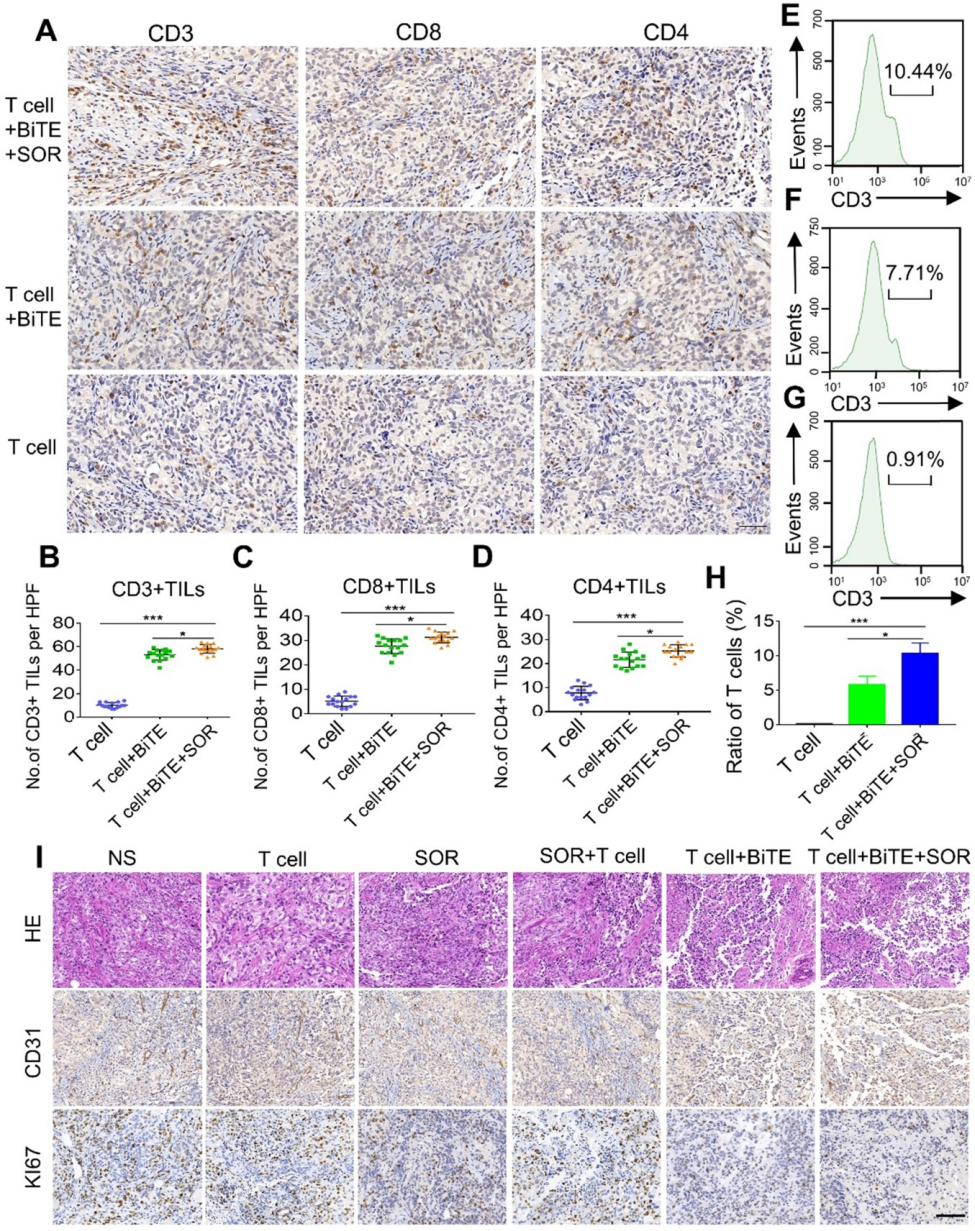
***In vivo* lymphocyte infiltrate and histology of tumors assessment in OC subcutaneous tumor models.** (**A**) Photomicrographs of representative tumors from T cell, T cell+BiTE and T cell+BiTE+SOR treatment groups depicting the immunohistochemistry for CD3, CD4 and CD8. Scare bar, 50 µm. (**B, C, D**) Quantification and comparison of CD3+, CD8+, CD4+ TILs from intact tumors in figure A. *P < 0.05, ***P < 0.001. (**E, F, G**) Tumor lymphocyte infiltrate was assessed using flow cytometry. Percentage of T cells from intact tumors that T cell+BiTE+SOR (E), T cell+BiTE (F) or the T cell (G) treated. (**H**) Histogram of the mean fluorescence intensity in figure E, F, G. *P < 0.05, ***P < 0.001. (**I**) Representative histology of tumors from different treatment groups with H&E staining (upper), CD31 expression (Middle) and Ki67 expression (lower). Scale bar, 100 µm.

The frequency of tumor infiltrating lymphocytes (TILs) was tested to evaluate whether activated T lymphocytes were recruited to the tumor site after treatment. We used IHC to evaluate the expressions of CD3, CD4, and CD8 in tumors after T cells, T cell+BiTE or T cell+BiTE+SOR treated (Figure 7A-D). Among these groups, BiTE combined with SOR treatment group exhibited a significantly higher number of CD3+, CD8+ and CD4+ TILs. Similarly, we further investigated the ratios of tumor‐infiltrating T cells by flow cytometry. The results suggested that the combined treatment group contained the highest percentage of T cells (Figure 7E-H). In addition, H&E staining showed the irregular structure in both the BiTE and the BiTE combined with SOR treatment group (Figure 7I). IHC staining of tumor sections showed that cell proliferation (Ki67 labeling) and vessel area (CD31 staining) decreased significantly in both the T cell+BiTE and T cell+BiTE+SOR treatment group (Figure 7I).

### Tumor lymphocytic infiltration was enhanced in combination therapy of B7H3×CD3 BiTE and SOR in OC intraperitoneal tumor xenograft models

**Figure 8:**
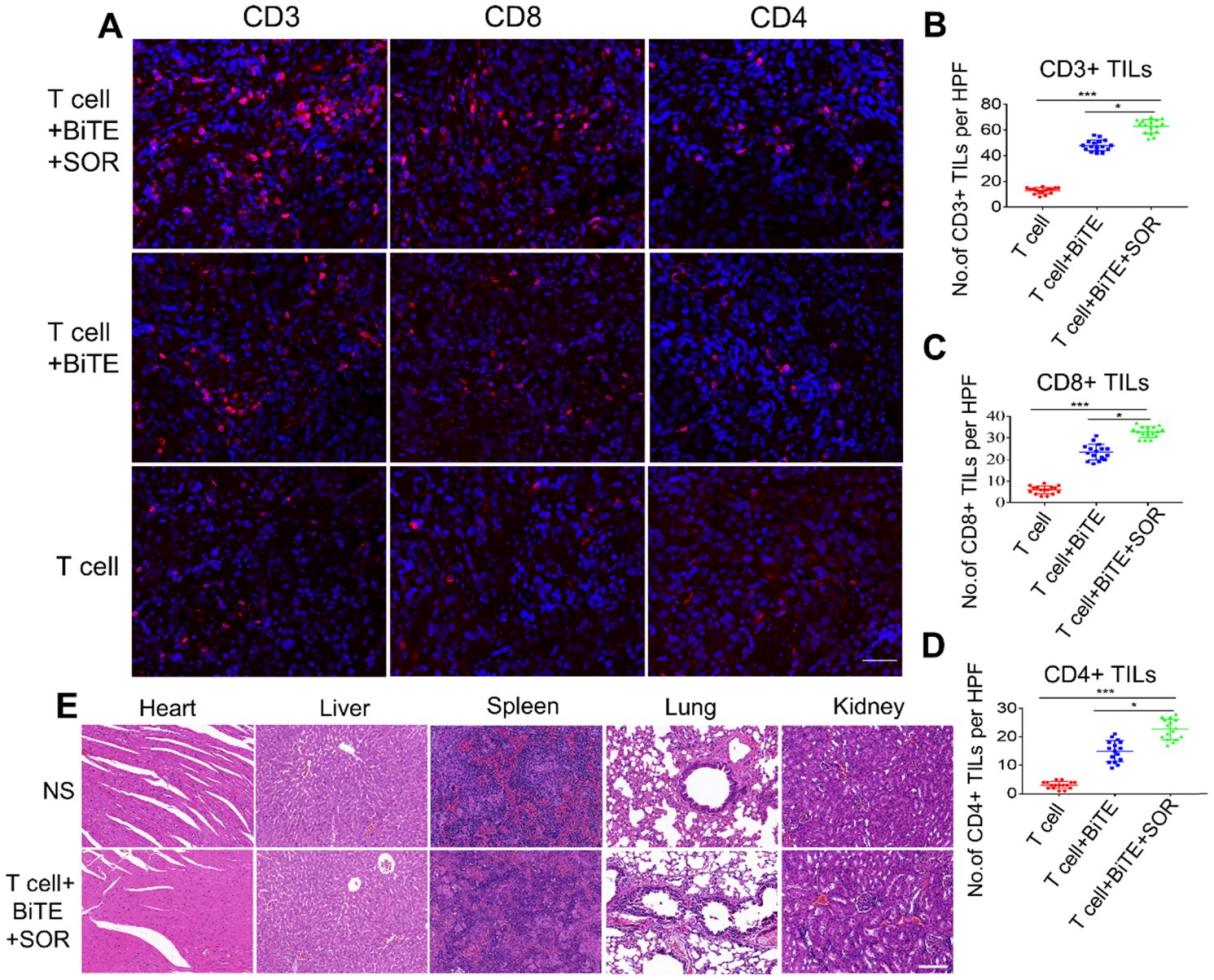
** Tumor lymphocytic infiltration and *in vivo* safety assessment in OC intraperitoneal tumor xenograft models.** (**A**) Tumor-infiltrating T-lymphocytes were tested by Fluorescence IHC for CD3, CD4 and CD8. Scare bar, 50 µm. (**B, C, D**) Statistical analysis of quantitative of CD3+, CD8+, CD4+ TILs from intact tumors that T cell+BiTE+SOR, T cell+BiTE and T cell treated. *P < 0.05, ***P < 0.001. (**E**) Preclinical safety assessment was evaluated by H&E staining. The pictures represent staining results of liver, spleen, kidney, heart and lung. Scare bar, 100 µm.

To further verify the *in vivo* infiltration of the lymphocytes, we also used an intraperitoneal model. We used fluorescent IHC to evaluate the expressions of CD3, CD4, and CD8 in tumors after T cell, T cell+BiTE or T cell+BiTE+SOR treated (Figure 8A-D). Relative to the T cell group, the T cell+BiTE+SOR had the highest number of lymphocytes, followed by the T cell+BiTE group. In addition, we performed a preliminary evaluation of *in vivo* safety of tumor-bearing mice. Microscopic observations did not find any lesions in the liver, spleen, kidney, heart and lung in OC mice from all treatment groups (Figure 8E).

## Discussion

Our data presented here support several novel findings. First, B7H3 was highly expressed in OC samples and it was correlated with reduced survival rates. Second, sorafenib was capable of suppressing the cell proliferation of OC without obvious inhibitory activity towards human T cells. Third, B7H3×CD3 BiTE successfully recruited T cells to mediate tumor killing of OC. Finally, combination therapy with sorafenib and B7H3×CD3 BiTE displayed an optimal antitumor activity of OC both *in vitro* and *in vivo*. Overall, this study showed the potential of the concurrent treatment of sorafenib and B7H3-redirecting antibodies in inducing efficient antitumor immune responses.

To date, sorafenib has already been used as an FDA-approved drug for renal cell carcinoma and hepatocellular carcinoma. Preclinical and clinic studies have demonstrated the antitumor efficacy of sorafenib in a broad range of human tumors 30. In this study, we found that sorafenib alone induced cell apoptosis and inhibited cell proliferation *in vitro*. In tumor-bearing mice, data suggested that sorafenib decelerated the growth of tumors through inducting of cell apoptosis, inhibiting of cell growth and angiogenesis. These data are consistent with the previous reports of sorafenib for the treatment of cancers with dysregulation of Ras/MEK/ERK pathway 31-33. These may expand its role in a broader spectrum of oncotherapy.

Several studies have shown sorafenib had an adverse effect on T cell proliferation at pharmacologic doses (10 μM) with minor impact at sub-pharmacologic doses (1-5 μM) 34, 35. However, a previous report demonstrated that sorafenib had no significant influence on the functions of human CAR-T at pharmacologic or the sub-pharmacologic dose. In this study, our Cyto Tell Red labeling method did not identify any inhibition effect on the growth of T cells. The discrepancy among these findings may, in part, be a result of different methods of T cell stimulation. Noticeable, recent studies revealed that sorafenib treatment enhanced functions of tumor-specific effector T cells and reduced the number of immunosuppressive cells and triggered the proinflammatory activity of TAMs 33, 36, 37. Our data also suggested a combination of sorafenib and the BiTE significantly promoted tumor infiltration in mice models and 3D tumorspheres. Taken together, these results reflect that sorafenib may be beneficial for T-cell-based immunotherapy.

The selection of appropriate target antigens in solid tumors is closely associated with the therapeutic effect of BiTE. A previous study showed effective antitumor responses in OC by targeting B7H3 via chimeric antigen receptor T cells, which is similar to our findings that B7H3-redirecting BiTE can significantly induce tumor killing *in vitro* and *in vivo* 20. Interestingly, treatment with sorafenib mediated a noticeable elevation of B7H3 expression at sub-pharmacologic doses (1-5 μM). However, the phenomenon disappeared when the concentration of sorafenib increased. Sorafenib is a multi-kinase inhibitor initially developed to inhibit the Raf1-kinase pathway. However, besides the RAF/MEK/ERK pathway, sorafenib also targets other multiple intracellular and cell surface kinases like VEGFR, PDGFR, FLt-3, RET, c-KIT, et al. 23. Numerous intracellular pathways will be altered after treatment with sorafenib. Thus, we cannot to conclude that if the increment of B7-H3 is through MEK-ERK pathway only from our limited pathway analysis data. While further research is definitely required to elucidate the underlying mechanism of B7H3 up-regulation, our current data support the feasibility of combination therapy with sorafenib and agents targeting B7H3.

In this article, two different animal xenograft models were selected to investigate the effectiveness of the combination treatment. Given that the technique of subcutaneous injection is straightforward, OC cell line SKOV3-Lucf cells were used to construct a subcutaneous transplanted tumor model. To further accurately simulate the aspects of tumor metastasis, particularly metastatic dissemination, the intraperitoneal tumor models are required as it can simulate the process of peritoneal dissemination and ascites formation in OC, and has generally been used to evaluate the treatment effect. Our data indicated that the therapeutic effect in intraperitoneal tumor model outperformed the subcutaneous tumor models. However, preclinical results in mouse models often failed to show efficacy in phase III clinical trials on patients with metastatic tumors 38. To better simulate the microenvironment and establish a more accurate relationship between preclinical studies and clinical outcomes, orthotopic model is more reliable.

In summary, the findings clearly indicate an abnormally high frequency of B7H3 expression in OC samples. Both sorafenib and B7H3×CD3 BiTE showed notable antitumor activities *in vitro* and *in vivo*. The combination treatment of the two agents achieved a remarkable synergistic therapeutic effect. Although more penetrating studies are needed to ascertain the mechanisms of the synergy, this study may shed light on future OC clinical trials using combination treatments with sorafenib and B7H3-redirecting BiTE.

## Supplementary Material

Supplementary figures and tables.

## Abbreviations

B7H3: B7 homolog three
BiTE: Bispecific T-cell engaging antibody
BLI: Bioluminescent imaging
E:T: effector-to-target
ELISA: enzyme linked immunosorbent assay
IHC: immunohistochemistry
mAb: monoclonal antibody
OC: ovarian cancer
SOR: sorafenib
scFv: single-chain antibody fragment
TCGA: The Cancer Genome Atlas
