# Supplementary material for: Combination therapy with B7H3-redirected bispecific antibody and Sorafenib elicits enhanced synergistic antitumor efficacy
Source: Theranostics. 2020 Aug 21;10(23):10498–512. doi: 10.7150/thno.49480 (PMC7482810; doi:10.7150/thno.49480)
Supplement: Supplementary file 1 — Supplementary figures and tables. [file thnov10p10498s1.pdf]

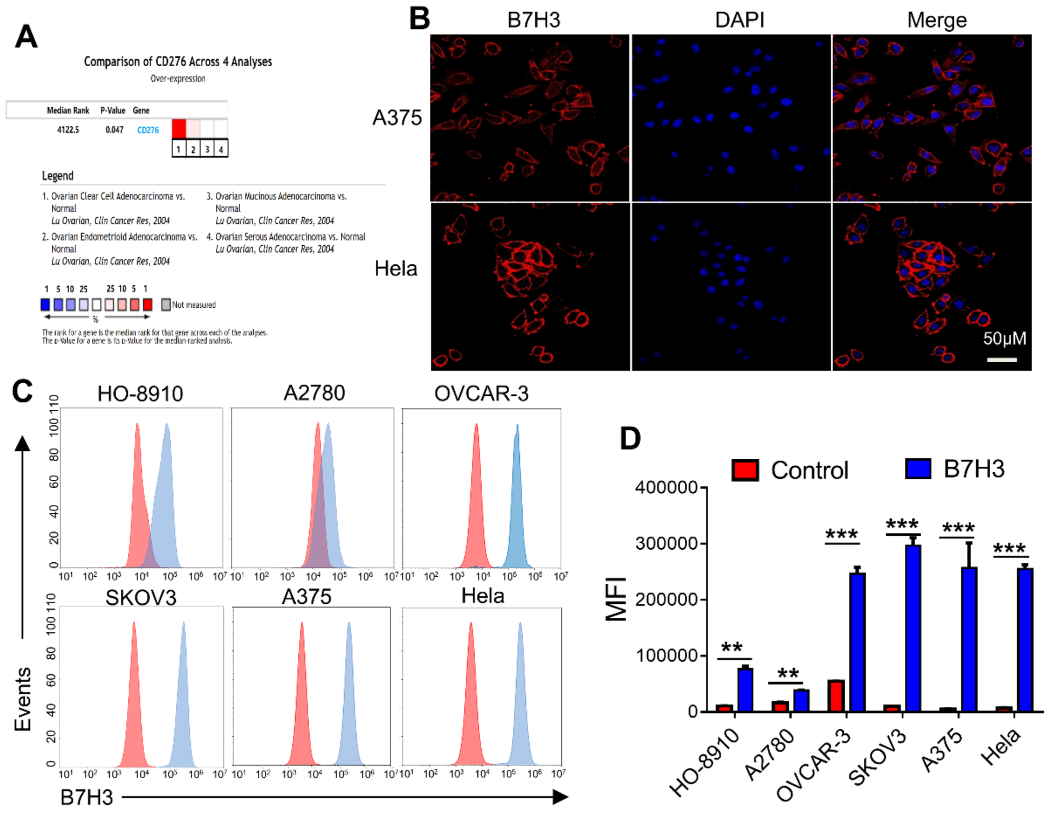

Supplementary Figure S1. Expression of the costimulatory molecule B7H3 in different cancer samples. (A) Expression of B7H3 in OC in the studies derived from the Oncomine database. (B, C) Flow cytometry and immunofluorescence staining patterns showed high expression of the costimulatory B7H3 in different cancer cell lines (HO-8910, A2780, OVCAR-3, SKOV3, A375, Hela). (D) Histogram of the mean fluorescence intensity in figure C. **P < 0.01, ***P < 0.001.

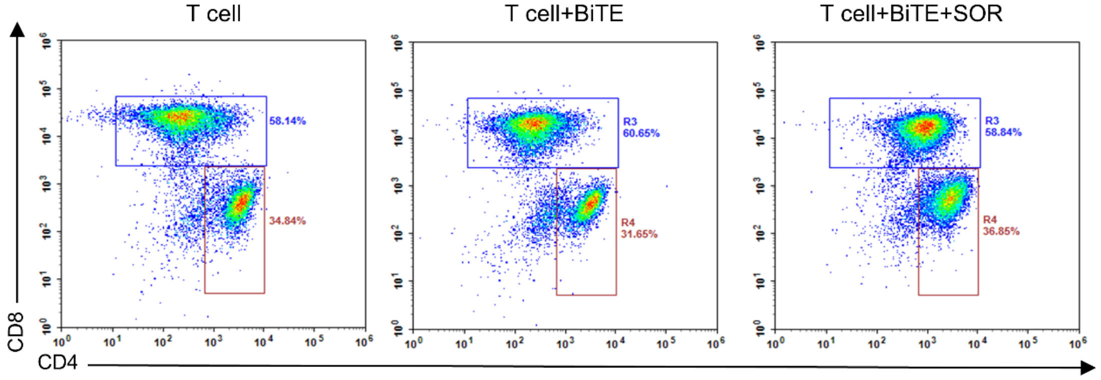

Supplementary Figure S2. The ratio of CD4 and CD8 positive T cell in coculture assay with tumor cells. Dot plot diagram of flow cytometry showing CD4+ and CD8+ percentage of human T cells after coculture with SKOV3 cells by adding 5 μM SOR alone or in combination with 5 μg/mL B7H3×CD3 BiTE.

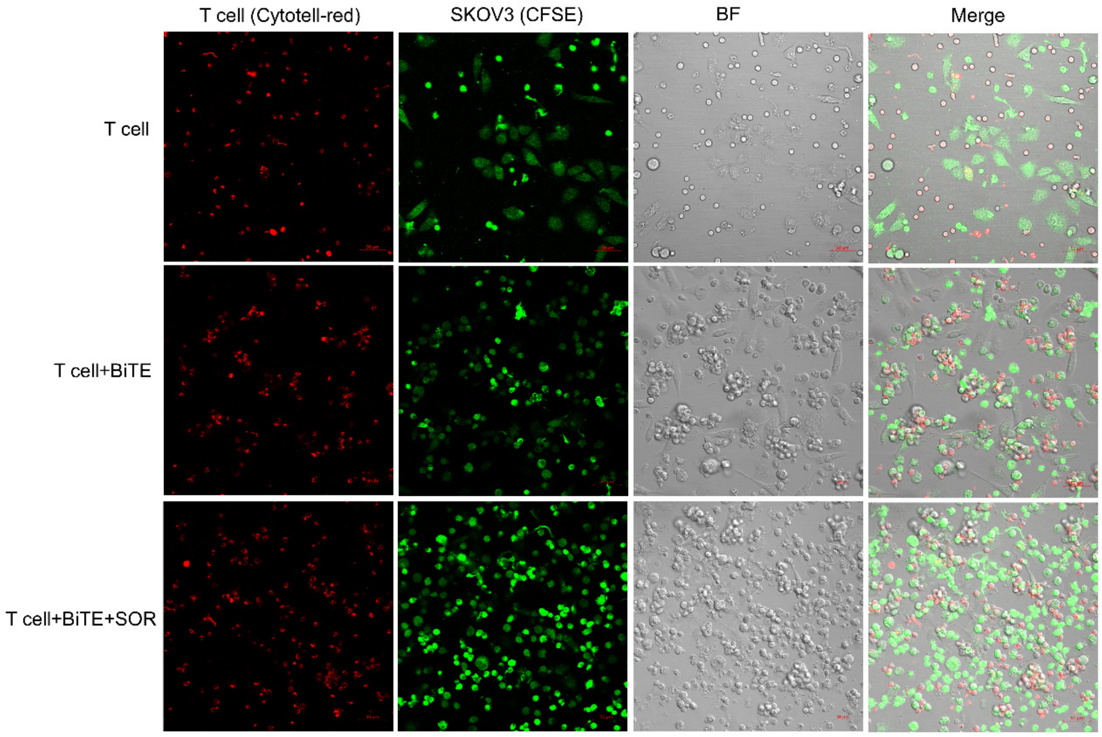

Supplementary Figure S3. The morphology of tumor cell lysis was analyzed using confocal microscopy. Target cells (SKOV3) and effector cells (T cell) were fluorescently labeled with CFSE and Cyto Tell Red, respectively(E:T=5:1). The observation was performed at 24 h after co-culture under the different condition.

The sequence of anti-CD3

Nucleic acid sequence:
gacatcaagctgcagcagtcaggggctgaactggccaggcctggggcttcagtgaagatgtcctgcaagacctctggctacaccttca
ccagatacaccatgcactgggtgaagcagaggcctggacaaggccttgagtggatcggatacattaacccttctagaggctatactaa
ctacaatcaaaagttcaaggacaaggccacattgactaccgacaagtcctccagcacagcctacatgcagctcagcagcctgacatct
gaggactctgcggtctattactgtgccagatattacgacgaccactattgcctggactactggggccaaggcaccacgctgaccgtcag
cagcgtggagggcggttcaggcggaagcggcgggagcggtggcagcggaggcgtggacgacatccagctgacccagagccca
gccatcatgagcgccagccccggcgagaaggtgaccatgacctgtagggccagctcaagtgtaagttacatgaactggtaccagcag
aagagcggtaccagcccaaagagatggatctacgacacatccaaggtggcttctggtgtgccatacagattcagcggtagcggtagc
ggtaccagctacagcctcaccatcagcagcatggaggctgaggacgccgccacctactactgccagcagtggagtagtaacccactc
acgttcggcgctgggaccaagctg

Amino acid sequence:
DIKLQQSGAELARPGASVKMSCKTSGYTFTRYTMHWVKQRPGQGLEWIGYINPSRG
YTNYNQKFKDKATLTTDKSSSTAYMQLSSLTSEDSAVYYCARYYDDHYCLDYWGQG
TTLTVSSVEGGSGGSGGSGGSGGVDDIQLTQSPAIMSASPGEKVTMTCRASSSVSYMN
WYQQKSGTSPKRWIYDTSKVASGVPYRFSGSGSGTSYSLTISSMEAEDAATYYCQQW
SSNPLTFGAGTKL
